# Lysosome trafficking is necessary for EGF-driven invasion and is regulated by p38 MAPK and Na+/H+ exchangers

**DOI:** 10.1186/s12885-017-3660-3

**Published:** 2017-10-04

**Authors:** Samantha S. Dykes, Joshua J. Steffan, James A. Cardelli

**Affiliations:** 1 Department of Microbiology and Immunology, Louisiana State University Health Sciences Center – Shreveport, Shreveport, LA 71130 USA; 2 Feist-Weiller Cancer Center, Louisiana State University Health Sciences Center- Shreveport, Shreveport, LA 71130 USA; 3 Department of Natural Sciences, Dickinson State University, 291 Campus Dr, Dickinson, ND 58601 USA; 4 Present Address: Department of Radiation Oncology, University of Florida, Gainesville, FL 32608 USA

**Keywords:** Lysosome, Trafficking, EGF, p38, NHE, Signaling, Invasion, 3D culture

## Abstract

**Background:**

Tumor invasion through a basement membrane is one of the earliest steps in metastasis, and growth factors, such as Epidermal Growth Factor (EGF) and Hepatocyte Growth Factor (HGF), stimulate this process in a majority of solid tumors. Basement membrane breakdown is one of the hallmarks of invasion; therefore, tumor cells secrete a variety of proteases to aid in this process, including lysosomal proteases. Previous studies demonstrated that peripheral lysosome distribution coincides with the release of lysosomal cathepsins.

**Methods:**

Immunofluorescence microscopy, western blot, and 2D and 3D cell culture techniques were performed to evaluate the effects of EGF on lysosome trafficking and cell motility and invasion.

**Results:**

EGF-mediated lysosome trafficking, protease secretion, and invasion is regulated by the activity of p38 mitogen activated protein kinase (MAPK) and sodium hydrogen exchangers (NHEs). Interestingly, EGF stimulates anterograde lysosome trafficking through a different mechanism than previously reported for HGF, suggesting that there are redundant signaling pathways that control lysosome positioning and trafficking in tumor cells.

**Conclusions:**

These data suggest that EGF stimulation induces peripheral (anterograde) lysosome trafficking, which is critical for EGF-mediated invasion and protease release, through the activation of p38 MAPK and NHEs. Taken together, this report demonstrates that anterograde lysosome trafficking is necessary for EGF-mediated tumor invasion and begins to characterize the molecular mechanisms required for EGF-stimulated lysosome trafficking.

**Electronic supplementary material:**

The online version of this article (10.1186/s12885-017-3660-3) contains supplementary material, which is available to authorized users.

## Background

Tumor cell invasion is driven by many factors, including cell surface receptor tyrosine kinases, which are often highly expressed or hyper-activated in cancers [1]. Epidermal growth factor receptor (EGFR) and hepatocyte growth factor receptor (c-Met) are two receptor tyrosine kinases known to contribute to tumor progression [2]. While both c-Met and EGFR drive tumor cell growth and invasion, many tumors exhibit EGFR-driven growth independent of c-Met activation. Binding of the epidermal growth factor (EGF) ligand to EGFR induces homo- or hetrodimerization of the receptor and activation of the kinase domain, ultimately leading to intracellular signaling events, including activation of protein kinase B (AKT), extracellular signal-regulated kinase (ERK), and p38 mitogen-activated protein kinase (MAPK). EGFR signaling cascades are known to regulate proliferation, cell survival, motility, and invasion (Reviewed in [3]). Moreover, EGFR expression and activity are increased in many solid tumors compared to normal adjacent tissues, and EGFR activation is known to increase invasiveness [4, 5].

Lysosomes are acidic organelles rich in proteases and hydrolases that function to degrade and recycle cellular proteins and other macromolecules. The activation and signaling of both the EGFR and c-Met receptor are regulated, in part, by lysosomal degradation [6, 7]. Abnormal receptor trafficking, organelle fusion, or lysosome integrity, will cause growth factor receptors to recycle back to the plasma membrane for continued signaling events in contrast to be degraded [8]. Thus, lysosomes normally provide tight control of receptor tyrosine kinase signaling; however, disruption of lysosomal function and/or location can promote tumor invasion.

In addition to regulating receptor tyrosine kinase signaling events, lysosomes can release proteases into the extracellular space causing extracellular matrix (ECM) degradation, a hallmark of invasive cancers [9–11]. One mechanism of lysosome secretion involves the movement (trafficking) of lysosomes to the cell periphery to promote fusion with the plasma membrane and subsequent extracellular release of lysosomal contents. Lysosome positioning and trafficking throughout the cell is mediated by the activity of kinesin and dynein motor proteins, which move organelles and other vesicles along microtubules and actin filaments to the cell periphery or inward toward the microtubule-organizing center (MTOC), respectively [12, 13]. In non-invasive cells, lysosomes are located in the perinuclear region. In contrast, lysosomes in invasive cells redistribute to the periphery and localize to invadopodia, or focalized sites of matrix degradation [14–18]. Interestingly, increased levels of the lysosomal protease cathepsin B can be found in the serum of cancer patients and inhibition of proteolysis slows tumor invasion in vitro [18–21].

Recent findings demonstrated that HGF/c-Met signaling induced lysosome redistribution to the periphery of tumor cells leading to increased secretion of the lysosomal protease cathepsin B. This anterograde (microtubule plus end or outward) lysosome trafficking was necessary for HGF/c-Met-mediated tumor cell invasion and activated c-Met stimulated anterograde lysosome trafficking via signaling through phosphoinositide-3-kinase (PI3K) and sodium/hydrogen exchangers (NHEs) [15, 17]. Since many solid tumors exhibit EGFR-driven growth independent of c-Met activation, this study investigates the role of EGF/EGFR signaling in anterograde lysosome trafficking.

In the present study, we demonstrate that EGF stimulation results in anterograde lysosome trafficking and that this lysosome trafficking event is necessary for EGF-mediated invasion. Anterograde lysosome trafficking was dependent upon NHE activity; however, unlike previously investigated stimulatory events, EGF-mediated lysosome trafficking was dependent on p38 MAPK. In addition to regulating lysosome trafficking, both NHE and p38 MAPK activity were required for EGF-mediated protease secretion and invasion in 3-dimenisional (3D) cell culture.

## Methods

### Cell culture

DU145 cells were purchased from ATCC (ATCC-HTB-81, Manassas, VA) and maintained in RPMI 1640 media (Mediatech, Corning, NY) supplemented with 10% Fetal Bovine Serum (FBS). HeLa cells were obtained from ATCC (ATCC-CCL-2) and maintained in DMEM media (Mediatech) supplemented with 10% FBS. Cells were grown at 37 °C in 5% CO_2_ and passaged upon reaching 75% confluence.

### Reagents and antibodies

Troglitazone, AG490, Bay11, SP600125, PD169316, and SB203580 were purchased from Cayman Chemicals (Ann Arbor, MI). Hepatocyte Growth Factor, SB202474, AG1478, U0126, and SU11274 were purchased from Calbiochem (San Diego, CA). SB239063 and LY294002 were obtained from Enzo Life Sciences (Farmingdale, NY). Epidermal Growth Factor and 5(N-Ethyl-N-isopropyl) amiloride (EIPA) were acquired from Sigma (St. Louis, MO). Antibodies recognizing total p38 MAPK and phosphorylated EGFR Y845, Met Y1234/1235, AKT S473, MAPK 44/42 T202/204, and p38 MAPK T180/Y182 were used at 1:1000 and supplied by Cell Signaling Technology (Beverly, MA). Antibodies recognizing total EGFR (1:1000), AKT1 (1:4000) and ERK 1/2 (1:4000) were obtained from Santa Cruz Biotechnology (Dallas, TX). The total c-Met (1:1000) antibody was purchased from Life Technologies (Carlsbad, CA). The α-tubulin antibody was purchased from NeoMarkers (Fremont, CA) and was used at 1:20,000. The LAMP-1 H4A3 antibody was supplied by the Developmental Studies Hybridoma Bank at the University of Iowa and was used at a 1:200 dilution for immunofluorescence. Matrigel, anti-EEA1, and anti-GM130 were obtained from BD Bioscience (San Jose, CA) and used at 1:100. DQ-collagen IV, Oregon Green or 635 Phalloidin (1:200) and mounting media containing DAPI plus SlowFade Gold reagent were obtained from Invitrogen Life Technologies (Grand Island, NY). Dylight 594 donkey anti-mouse was purchased from Jackson Immuno Research (West Grove, PA) and used at 1:200. Secondary antibodies (HRP- conjugated anti-mouse and anti-rabbit) for western blot were purchased from GE Healthcare, Pittsburgh, PA and used at 1:5000. Since a majority of the pharmacological inhibitors were solubilized in DMSO, a DMSO concentration of 0.1% was in contact with the cells and used as a control in all pharmacological inhibitory experiments.

### Immunofluorescence

Experiments conducted in 2-diminesional cell culture, cells were seeded at ~50% confluence on glass cover slips. Following treatment, cells were fixed with ice cold 4% paraformaldyhide (PFA) pH 7.2 for 20 min. Cells were washed twice with phosphate buffered saline (PBS) then incubated for 1 h with primary antibody diluted in 0.25% bovine serum albumin (BSA) and 0.1% Saponin in PBS (BSP). After incubation with primary antibody, cells were washed twice with PBS and incubated with fluorescently conjugated secondary antibody diluted in BSP for 1 h. To visualize the cytoskeleton, cells were incubated with phalloidin diluted in BSP for 20 min. Cells were then washed three times in PBS and mounted using DAPI with Slow Fade Gold reagent. Images were taken using an Olympus UPlanFl 40X/0.75 objective on an Olympus BX50 microscope, utilizing a Roper Scientific Sensys Camera, and MetaMorph software. Images were pseudocolored and merged using ImageJ. For 3-dimensional immunofluorescence of LAMP-1, all reagents were warmed to 37 °C. Cultures were fixed with 4% PFA for 20 min then quenched with 100 mM glycine in PBS for 10 min. Cells were then washed 2X with PBS and permeabilized/blocked for 30 min with 10% donkey serum and 1% F(ab)_2_ Fragment anti-mouse (Jackson IR, West Grove, PA) diluted in BSP. Cells were washed 2X in PBS with the remainder of the protocol remaining the same as for 2-dimensional immunofluorescence. Images were taken using a HCX Plan Apo 63X/1.4–0.6 oil objective on a Leica TCS SP5 microscope utilizing Leica LAS AF software.

### 3D culture

3D cultures supplemented with DQ-collagen IV were prepared using a modification of a previously described protocol [22]. Briefly, 120 μL ice cold Matrigel was supplemented with 25 μg/mL DQ-collagen IV, plated on coverslips, and allowed to solidify at 37 °C for 15 min. 1X10^5^ cells were diluted in media containing serum and plated on top of the solidified extracellular matrix for two days to allow for colony formation. Once multicellular colonies were visualized, the media was replaced with serum free media containing inhibitors and/or growth factor for 48 h. Colonies were then fixed for 30 min with 37 °C 4% PFA and washed twice with warm PBS. After staining and imaging, images were analyzed for extracellular DQ-collagen IV signal using Image J. Briefly, a mask was generated to include the area of the phalloidin staining. This area was subtracted from the DQ-collagen IV signal using Image Calculator. Remaining extracellular DQ-collagen IV signal was recorded as integrated density and displayed as arbitrary units.

### Western blot analysis

Performed as previously described [16].

### Lysosome analysis

LysoTracker software was a generous gift from Meiyappan Solaiyappan at Johns Hopkins University [23]. This program was used to analyze the distance of fluorescently labeled lysosomes from the nucleus border. Twenty-five representative cells spanning three independent experiments were analyzed for each experimental condition.

### Transwell invasion assay

50 μL of a 1:5 dilution of Matrigel in serum free RPMI was plated on Costar Transwell Permeable Support inserts with 8.0 μm pores and allowed to solidify at 37 °C for two hours. Matrigel was re-hydrated with 50 μL serum free media for an additional 30 min at 37 °C. 1X10^4^ cells including pharmacological inhibitors and/or EGF were seeded in a total volume of 100 μL on top of the insert and allowed to invade for 48 h. Growth factor and inhibitor treatments were maintained in serum free media for the duration of the experiment. Transwell membranes were then fixed with 4% PFA for 20 min and stained with crystal violet for 20 min. Transwell inserts were washed with PBS and cells remaining on the top of the insert were removed using a cotton swab. Five representative 10X fields were counted from three independent experiments.

### Wound healing and scattering assays

Cells were plated in 12 well dishes and grown to a confluent monolayer. The monolayer was then scratched using a p200 pipette tip. Cells were washed twice with PBS to remove any debris and then treated with serum free media containing the inhibitor and/or growth factor. Cells were allowed to migrate into the wound for 24 h. One well was scratched immediately before fixation and served as a *T* = 0 scratch control (indicated by yellow lines). For scattering assays, cells were plated at 40% confluence and cultured under the indicated conditions for 16 h. Cells were then fixed with 4% PFA for 20 min and stained with 488 phalloidin diluted in BSP for 20 min. Cells were imaged using a Nikon Eclipse TE300 inverted microscope, Photometrics CoolSNAPfx monochrome 12-bit camera and a 4X (wound healing) or 10X (scattering) CFI Plan APO objective. Cell scattering was quantitated by counting the number of scattered cells per total objects in each field from three independent experiments. Wound healing was assessed by tracing the borders of the wound and calculating the wounded area with Image J software.

### Densitometry analysis

ImageJ software was used for western blot quantification. The ratio of the intensity of each protein band to its corresponding tubulin load control was calculated and graphed.

### Statistics

Significance was determined using a Two-Tailed, Mann-Whitney T-test utilizing GraphPad Software, Prism 3.0. A significant difference resulted when *p* < 0.05. All error bars represent the standard error of the mean.

## Results

### Different downstream signaling events regulate HGF- and EGF-induced cell scattering

**Fig. 1:**
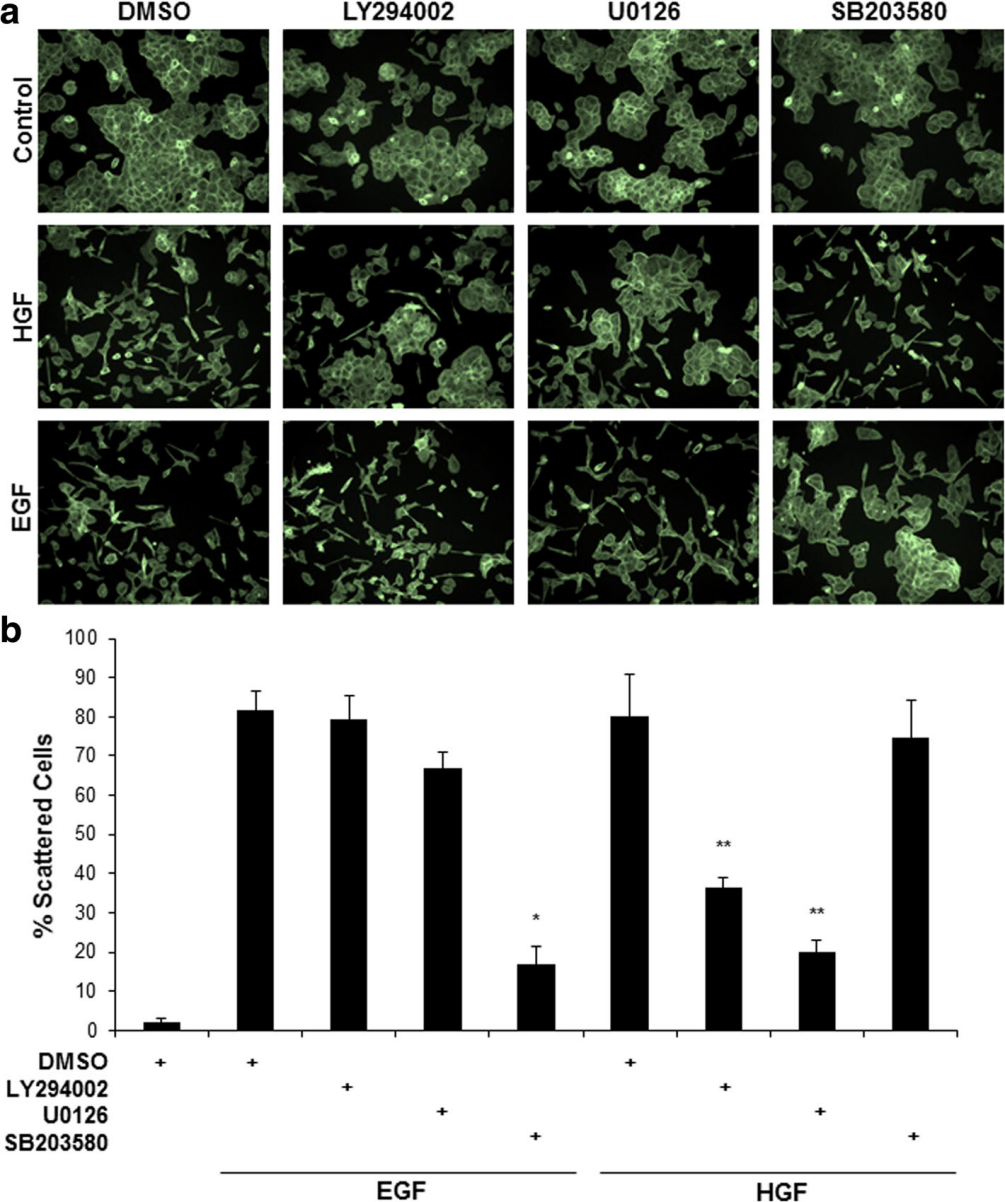
HGF and EGF mediate cell scattering via different downstream signaling pathways. **a** DU145 cells were pretreated with 10 μM of the indicated inhibitors or 0.1% DMSO for 30 min prior to stimulation with 100 ng/mL EGF or 33 ng/mL HGF for 16 h. Cells were fixed and stained with phalloidin. Cell scattering was imaged in 10X fields, *N* = 3. **b** Represents % scattered cells analyzed from three independent experiments. * = *p* < 0.001 compared to EGF control and ** = *p* < 0.001 compared to HGF control

Cell scattering is a morphological readout for in vitro motility and is often associated with tumor cell response to growth factor stimulation. Signaling through both the HGF/c-Met and EGF/EGFR is a potent inducer of cell scattering in the DU145 prostate cancer cell line [24–27]. We used several specific inhibitors to test whether these two receptor tyrosine kinases utilized similar down-stream signaling cascades to regulate scattering. DU145 cells were treated with specific inhibitors of PI3K/AKT (LY29004) [28], MEK/ERK (U0126) [29] or p38 MAPK (SB203580) [30] then stimulated with either HGF or EGF. All inhibitors were used at 5 or 10 μM unless otherwise noted. These inhibitor concentrations have been previously shown by our lab to be pathway specific and not inhibit other signaling pathways under the conditions of this study [16]. Cells were fixed and stained with FITC-labeled phalloidin to visualize F-actin and the percent of scattered cells was analyzed for each experimental condition (Fig. 1a; quantified in Fig. 1b). Control-treated cells assumed a cobblestone morphology which lost cell-cell adhesions upon treatment with growth factors. Inhibition of PI3K/AKT or MEK/ERK inhibited HGF-mediated scattering as previously described [17]. However, only p38 inhibition, and not inhibition of PI3K/AKT or MEK/ERK, blocked EGF-mediated cell scattering. This suggests that HGF/c-Met and EGF/EGFR regulate cell motility via different downstream pathways and that p38 MAPK activity is necessary for EGF/EGFR-mediated scattering.

### EGF/EGFR signaling results in anterograde lysosome trafficking independently of HGF/c-met signaling

**Fig. 2:**
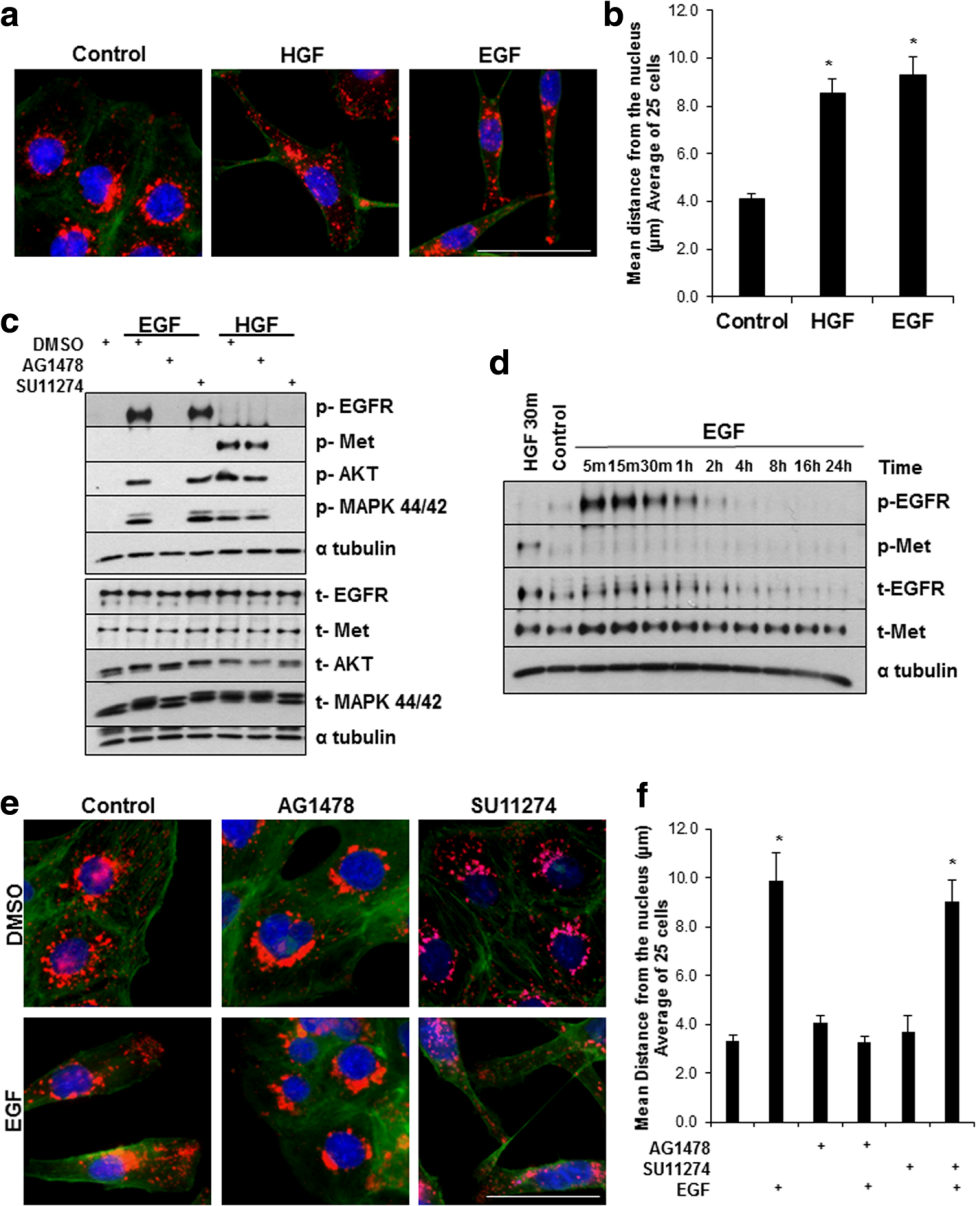
EGF-stimulated lysosome trafficking is due to EGFR activation and not crosstalk with c-Met. **a** DU145 cells were treated with 100 ng/mL EGF or 33 ng/mL HGF for 16 h then stained for LAMP-1 (red), actin (green) and DAPI (blue). Scale bar represents 30 μm, *N* = 3. **b** Quantification of lysosome distribution for 25 cells; mean values are shown. * = *p* < 0.05 compared to control. **c** DU145 cells were treated for 2 h with 10 μM AG1478 or SU11274 prior to stimulation with 100 ng/mL EGF or 33 ng/mL HGF for 10 and 30 min, respectively. Total protein lysates were harvested and analyzed by western blot. **d** Cells were stimulated with 33 ng/mL HGF for 30 min or 100 ng/mL EGF over time. Total protein lysates were harvested and analyzed via western blot. **e** DU145 cells were treated with 10 μM AG1478 or 5 μM SU11274, for 2 h then stimulated with 100 ng/mL EGF for 16 h. Cells were then fixed and stained for LAMP-1 (red), DAPI (blue), and phalloidin (green). Scale bar represents 30 μm, *N* = 3. **f** Quantification of lysosome distribution of 25 cells per condition. Error bars represent standard error of the mean. * = *p* < 0.05 compared to DMSO control

In addition to stimulating cell motility/scattering, HGF has been reported to redistribute lysosomes from the perinuclear region to the cell periphery and this lysosome redistribution is necessary for HGF-mediated invasion [15, 17]. We therefore asked whether EGF stimulation would similarly cause anterograde lysosome trafficking. DU145 cells were treated with EGF or HGF and then fixed and stained for lysosome-associated membrane protein-1 (LAMP-1) (red), actin (green), and DAPI (blue) (Fig. 2a; quantified in Fig. 2b). Similar to what was observed with HGF treatment, EGF stimulation resulted in anterograde trafficking of LAMP-1 positive lysosomes to actin rich cellular protrusions.

Several studies suggest that c-Met and EGFR undergo crosstalk and can transactivate each other; raising the possibility that EGF stimulation drives lysosome trafficking through c-Met transactivation [31–33]. To test whether EGFR transactivates c-Met, DU145 cells were first pre-treated with the c-Met inhibitor SU11274 [34] or the EGFR inhibitor AG1478 [35] and then stimulated with HGF or EGF. Western blot analysis revealed that HGF specifically activated c-Met signaling, which was not reduced in the presence of the EGFR inhibitor. Additionally, EGF activated EGFR and downstream EGFR signaling was not depleted under conditions of c-Met inhibition (Fig. 2c; quantified in Additional file 1: Figure S1). Dulak et al. suggested that EGF signaling results in c-Met activation at later time points [31]. Therefore, we treated cells with EGF over a 24-h time period and probed for EGFR and c-Met activation by western blot (Fig. 2d; quantified in Additional file 1: Figure S1). No increase in c-Met phosphorylation was observed at early or late timepoints post EGF stimulation, suggesting that there was no EGFR/c-Met signaling crosstalk in our system. In order to assess whether EGF-stimulated anterograde lysosome trafficking is EGFR specific, we treated cells with the EGFR inhibitor AG1478 or the c-Met inhibitor SU11274 in the presence or absence of EGF and observed the redistribution of LAMP-1 positive vesicles (red) by immunofluorescence microscopy (Fig. 2e; quantified in 2f). EGF-mediated anterograde lysosome trafficking was blocked by the addition of the EGFR inhibitor, but not the c-Met inhibitor. Together, these data suggest that EGF/EGFR signaling stimulates anterograde lysosome trafficking and this is not due to crosstalk with or transactivation of c-Met.

### Early endosomes, mitochondria, and the Golgi do not undergo anterograde trafficking in response to EGF stimulation

To examine whether other organelles redistribute to the periphery in response to EGF, cells were stimulated with EGF for 16 h then stained for markers of early endosomes, mitochondria, or the *cis-*golgi (Additional file 2: Figure S2). Organelle distribution relative to the nucleus was observed using immunofluorescence microscopy. EEA1 positive early endosomes were mostly diffuse throughout the cytoplasm, and did not re-localize to the cell periphery upon stimulation with EGF. Moreover, mitochondria and the *cis*-Golgi remained closely localized near the nucleus in both control and EGF treated cells. Thus, of the tested organelles, only LAMP-1 positive lysosomes underwent anterograde trafficking in response to EGF stimulation.

### Na+/H+ exchangers regulate EGF-mediated peripheral lysosome trafficking and invasion

**Fig. 3:**
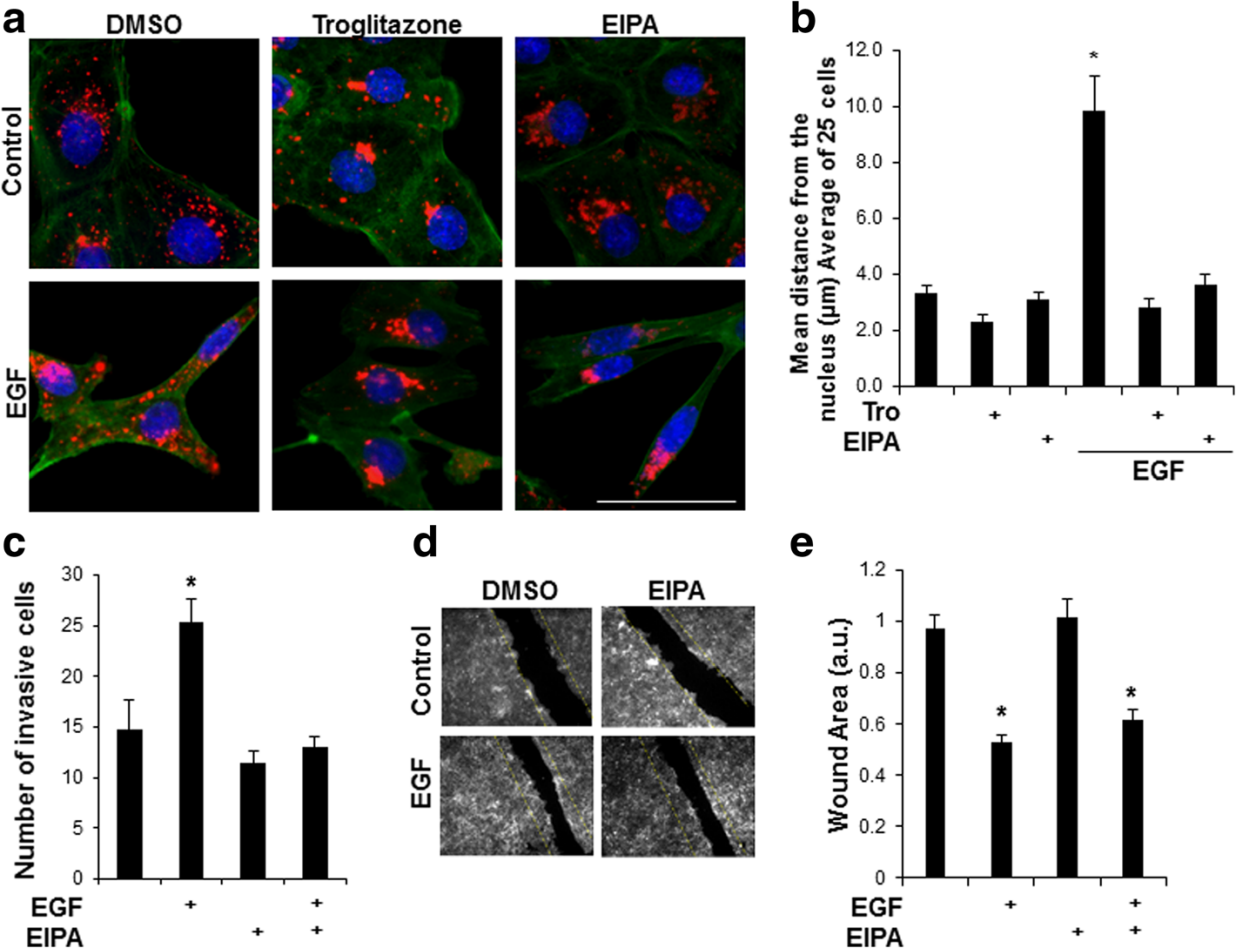
NHE Activity is necessary for EGF-mediated lysosome trafficking and invasion, but not overall cell motility. **a** DU145 cells were treated with 0.1% DMSO, 25 μM EIPA or 10 μM Tro for 2 h prior to a 16 h stimulation with 100 ng/mL EGF. Cells were then stained for LAMP-1 (red), phalloidin (green), and DAPI (blue). Scale bar represents 30 μm, *N* = 3. **b** Represents mean lysosome distribution of 25 cells; * = *p* < 0.05 vs. control. **c** DU145 were treated with 25 μM EIPA or 100 ng/mL EGF and allowed to invade through a 1:5 dilution of Matrigel for a 48 h boyden chamber invasion assay *N* = 3. The number of invasive cells were counted; * = *p* < 0.05 vs. control. **d** Confluent monolayers of DU145 cells were scratched with a p200 pipette tip and treated with DMSO or 25 μM EIPA for two hours prior to treatment with or without 100 ng/mL EGF. Cells were allowed to migrate into the wound for 24 h prior to fixation with 4% PFA and phalloidin staining. Representative 4X fields are shown, *N* = 3. Yellow lines indicate width of the initial p200 scratch. **e** Quantification of wound area from data in panel D. Error bars represent standard error of the mean. *p < 0.05 vs. control. (a.u. = arbitrary units)

Previous studies characterized NHEs as key regulators of anterograde lysosome trafficking in response to HGF stimulation [16, 17]. EGF stimulation is also known to activate plasma membrane NHEs [36, 37], raising the possibility that NHEs also regulate anterograde lysosome trafficking in response to EGF stimulation. To test this, we treated DU145 cells with 5-(N-ethyl-N-isopropyl)-Amiloride (EIPA), a general NHE inhibitor, or Troglitazone (Tro), an PPARγ agonist that we previously characterized as having a potent inhibitory effect on NHE function, in the presence or absence of EGF [14]. Cells were fixed and stained for LAMP-1 (red), actin (green), and DAPI (blue) (Fig. 3a; quantified in 3b). NHE inhibition with either EIPA or Tro prevented EGF-mediated anterograde lysosome trafficking. Similarly, EIPA treatment also prevented EGF-stimulated lysosome trafficking in HeLa cells (Additional file 3: Figure S3).

We next investigated whether juxtanuclear lysosome aggregation would prevent EGF-stimulated invasion or cell motility. Cells were stimulated with EGF in the presence or absence of EIPA and allowed to invade through a Matrigel-coated Boyden chamber. Cells were fixed and stained with crystal violet and the number of invasive cells were counted (Fig. 3c). Under conditions where lysosomes were clustered in perinuclear region as a result of EIPA treatment, EGF-stimulated invasion was reduced to levels comparable to that of control cells. Conversely, when cells under these same treatment conditions were assayed for cell motility using a scratch wound healing assay (Fig. 3d; quantified in Fig. 3e), NHE inhibition and prevention of lysosomal anterograde trafficking did not reduce overall cell motility. Therefore, the reduction of invasion upon EIPA treatment was not due to a reduction in overall cell motility. These results suggest that NHE inhibition and anterograde lysosome trafficking are necessary for EGF-mediated lysosome trafficking and invasion, but have no effect on overall cell motility.

### p38 MAPK activity is necessary for EGF mediated anterograde lysosome trafficking

**Fig. 4:**
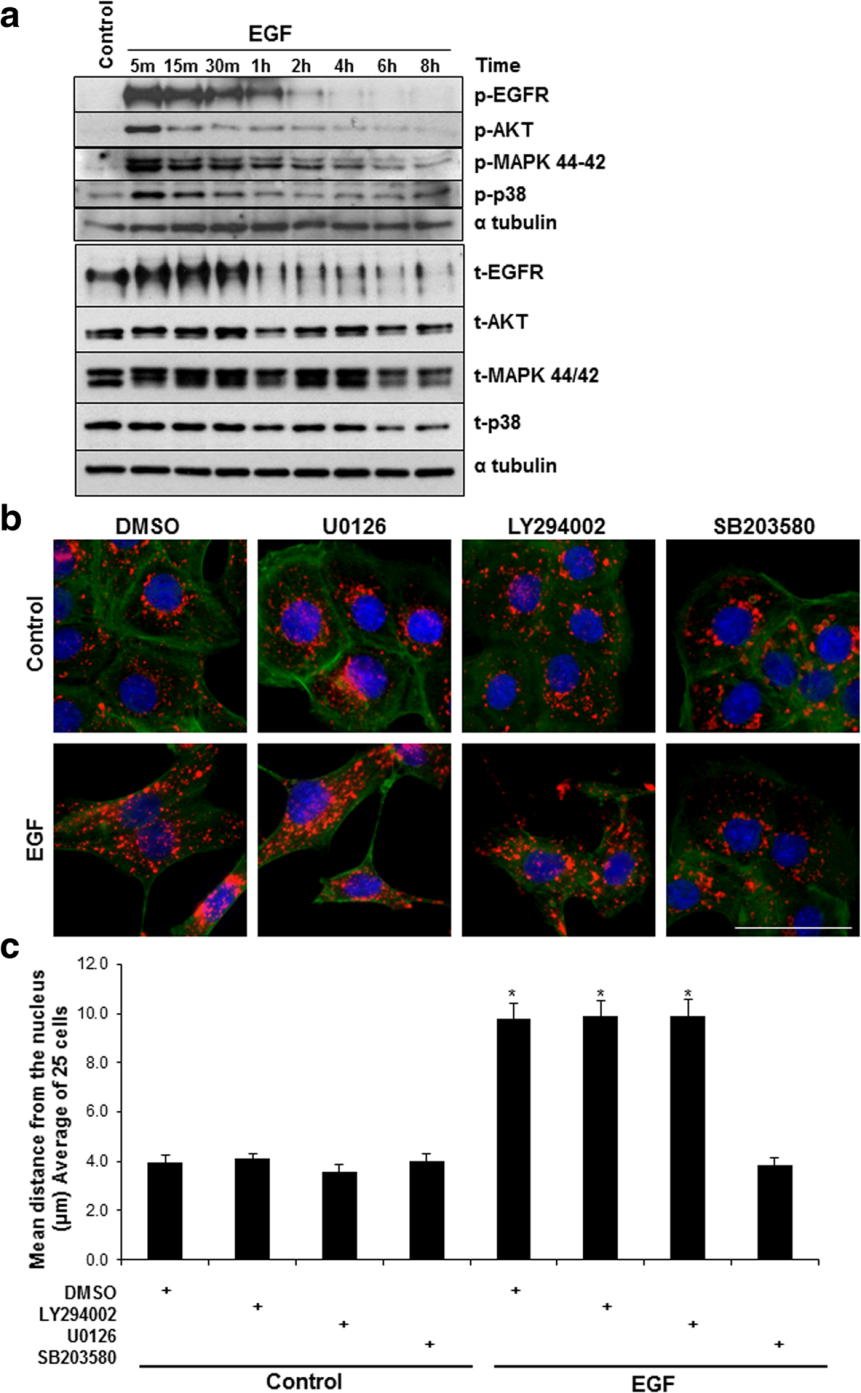
Small molecule inhibition of p38, but not PI3K or ERK, blocks EGF stimulated lysosome trafficking. **a** DU145 cells were stimulated with 100 ng/mL EGF over time. Total cell lysates were harvested and western blot analysis was performed. **b** Cells were treated with 10 μM of the MAPK inhibitor, U0126, the PI3K inhibitor, LY294002, or the p38 inhibitor SB203580 for 2 h prior to 16 h 100 ng/mL EGF treatment. Cells were fixed in 4% PFA and stained for LAMP-1 (red), phalloidin (green), and DAPI (blue). Scale bar represents 30 μm, *N* = 3. **c** Quantification of lysosome distribution of 25 cells per treatment. Error bars represent standard error of the mean. * = *p* < 0.05 vs. respective control treatments

We identified p38 MAPK as a key regulator of EGF-mediated cell scattering (Fig. 1), and questioned whether p38 MAPK activity also controlled EGF-mediated anterograde lysosome trafficking. To identify which signaling pathways were activated in response to EGF treatment in our system, DU145 cells were stimulated with EGF over time and assayed for levels of total or phosphorylated EGFR, ERK, AKT, and p38 MAPK by western blot (Fig. 4a; quantified in Additional file 4: Figure S4). EGF/EGFR activation results in the phosphorylation and activation of all tested downstream signaling proteins to varying degrees. To assess whether any of these downstream signaling components regulated EGF-induced lysosome trafficking, cells were pretreated with specific inhibitors of MEK/ERK (U0126), PI3K/AKT (LY294002) or p38α/β (SB203580) followed by stimulation with EGF. Cells were fixed and stained for LAMP-1 (red), actin (green), and DAPI (blue). Immunofluorescence microscopy revealed that p38 inhibition, but not inhibition of PI3K/AKT or MEK/ERK blocked EGF-mediated anterograde lysosome trafficking (Fig. 4b; quantified in Fig. 4c). Inhibition of p38 MAPK also blocked EGF-driven anterograde lysosome trafficking in HeLa cells (Additional file 3: Figure S3). To further confirm the involvement of p38 MAPK in the process of EGF-mediated anterograde lysosome trafficking, we used two additional p38 inhibitors, PD169316 and SB239063. SB202474 is an inactive analog of SB203580 and functions as a negative control. DU145 PCa cells were treated with the various p38 inhibitors in the presence or absence of EGF and lysosome positioning was assessed by immunofluorescence of LAMP-1 (red), actin (green), and DAPI (blue) (Additional file 5: Figure S5A). Treatment with either PD169316 or SB239063 prevented EGF-mediated anterograde lysosome trafficking. However, treatment with the inactive analog SB202474 failed to inhibit EGF-mediated lysosome trafficking, and LAMP-1 positive vesicles (red) were found out near the cell periphery (arrows) similar to what was seen with EGF treatment alone. In order to assess whether these p38 inhibitors were working, cells were pre-treated with each p38 inhibitor and then stimulated with EGF. Parallel western blot analysis revealed that all p38 inhibitors blocked EGF-mediated phosphorylation of p38, while the inactive analog (SB202474) did not (Additional file 5: Figure S5B). We also tested whether other downstream signaling pathways were involved in EGF-mediated anterograde lysosome trafficking. Cells were treated with inhibitors of Janus kinase-2 (JAK2, AG490), c-Jun N-terminal kinase (JNK, SP600125), or nuclear factor-κB (NFκB, Bay11) in the presence or absence of EGF and position of LAMP-1 positive vesicles (red) was analyzed by immunofluorescence (Additional file 5: Figure S5C). Inhibition of JAK, JNK, or NFκB did not prevent EGF-mediated anterograde lysosome trafficking. Collectively, these data indicated that p38 MAPK activity is necessary for EGF-mediated lysosome redistribution.

### EGFR signaling is not reduced in the presence of p38 MAPK inhibitors

**Fig. 5:**
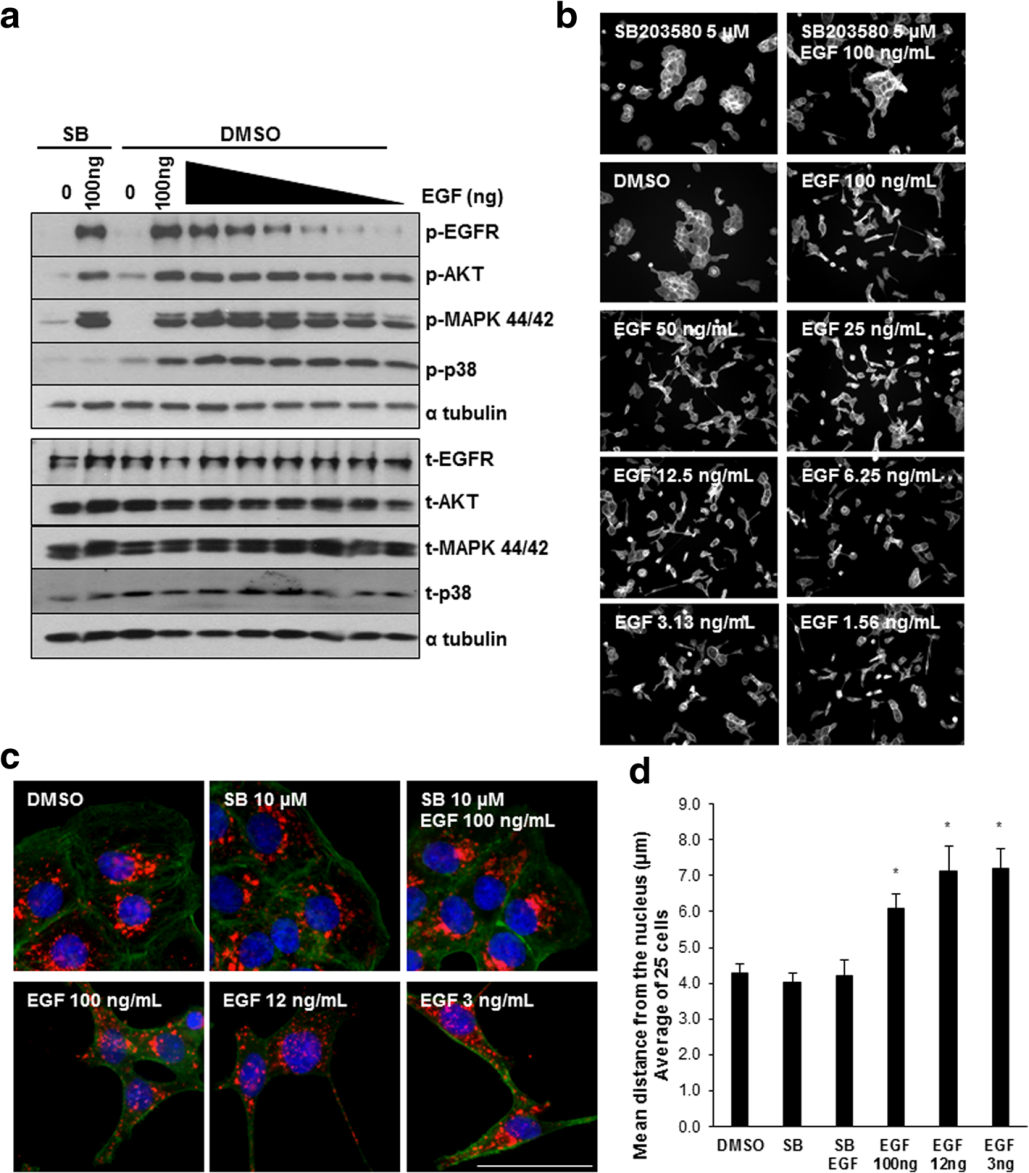
p38 inhibition does not block EGFR activation or signaling. **a** DU145 cells were treated with 10 μM SB203580 or 0.1% DMSO for 30 min prior to stimulation with varying concentration of EGF for 10 min. Whole cell lysates were collected and assessed by western blot. **b** Cells were treated with the indicated concentrations of SB203580 and EGF for 16 h. Cells were fixed and stained with phalloidin. Representative 10X images are shown, *N* = 3. **c** Cells were treated with the indicated concentrations of SB203580 or EGF for 16 h. Cells were fixed and stained for LAMP-1 (red), phalloidin (green), and DAPI (blue), *N* = 3. Scale bar represents 30 μm. **d** Quantification of lysosome distribution of 25 cells per treatment. Error bars represent standard error of the mean. * = *p* < 0.05 vs. DMSO

Previous reports suggest that EGFR does not effectively internalize or signal in the absence of p38 MAPK activity [38–41]. If EGFR is not signaling properly, this may be one explanation for the inhibition of cell scattering and anterograde lysosome trafficking seen upon p38 inhibition. To assess EGFR signaling, cells were treated with 100 ng/mL EGF in the presence or absence of the p38 inhibitor SB203580 or with decreasing concentrations of EGF and assessed by western blot (Fig. 5a; quantified in Additional file 6: Figure S6). Treatment with SB203580 blocked EGF-mediated p38 activity, but had no effect on levels of phosphorylated EGFR, ERK, or AKT (lane 2, Fig. 5a). This suggests that the PI3K/AKT and MEK/ERK signaling pathways are not suppressed as a result of off target effects of SB203580. Downstream signaling was maintained at comparable levels across a range of EGF concentrations (100 ng/mL- 3 ng/mL) (lane 4–10, Fig. 5a), even though receptor activation was reduced at the lower concentrations. We applied the same treatment conditions to a scattering assay (Fig. 5b) and found that DU145 cells still scattered with treatment of EGF as low as 1.56 ng/mL. Cells were then treated with vehicle, SB203580, SB203580 plus EGF, or varying concentrations of EGF and stained for actin (green), LAMP-1 (red) and DAPI (blue). Lysosome redistribution to the periphery still occurred in cells treated with 3 ng/mL EGF (Fig. 5c; quantified in Fig. 5d). Collectively these data support the idea that p38 inhibition does not significantly alter EGFR signaling in our system and that DU145 cells still undergo downstream signaling, scattering, and anterograde lysosome trafficking in response to very low levels of EGFR activation. Therefore, the loss of p38 activity results in the inhibition of EGF-driven anterograde lysosome movement, and this is not due to a reduction in overall EGFR signaling.

### EGF stimulates anterograde lysosome trafficking and protease secretion in 3D culture

**Fig. 6:**
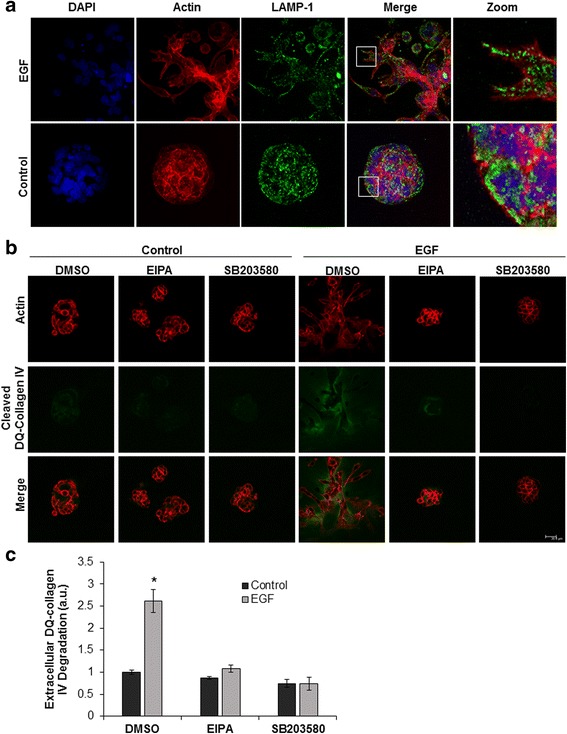
EGF treatment stimulates anterograde lysosome trafficking, protease secretion, and invasion in 3D culture. **a** DU145 cells were cultured on Matrigel in the presence or absence of 100 ng/mL EGF. Cells were fixed and stained for DAPI (blue), actin (red), and LAMP-1 (green). Representative 63X confocal images are shown, *N* = 3. **b** DU145 cells were plated in Matrigel and 25 μM DQ-Collagen IV for 48 h. Cells were then treated with 25 μM EIPA, 10 μM SB203580, or stimulated with 100 ng/mL EGF for 48 h. Cells were then fixed and stained with phalloidin (red). Cells and cleaved DQ-Collagen IV (green) were imaged using confocal microscopy. Representative 40X images are shown and scale bar represents 30 μm, *N* = 3. **c** Quantification of extracellular DQ-collagen IV fluorescence from panel B. Error bars represent standard error of the mean. * = *p* < 0.01 compared to control

Cell culture on a 2D plastic or glass surface does not accurately represent the 3-dimensional (3D) architecture of a solid tumor. Recent advances in 3D culture suggest that cell phenotypes vary greatly between cells cultured in 2D vs. 3D environments [42]. We observed that EGF stimulation resulted in anterograde lysosome trafficking in 2D culture (Fig. 2), and queried whether this same phenotype was maintained in cells grown in 3D culture. To address this, we cultured DU145 cells on Matrigel in the presence or absence of EGF. Cells were fixed and stained for DAPI (blue) actin (red), and LAMP-1 (green) and images were collected using confocal microscopy (Fig. 6a). Control-treated DU145 cells formed spheroid-like colonies, indicative of non-invasive cells. In contrast, EGF-treated cells formed irregular colonies and many cells had a mesenchymal morphology, suggesting that EGF stimulates an invasive phenotype in 3D culture. Additionally, LAMP-1 positive vesicles were localized to actin rich cellular protrusions along the leading edge of EGF-treated cells.

Our lab has previously characterized anterograde lysosome trafficking events as being necessary for acidic extracellular pH and HGF-mediated invasion and cathepsin B secretion in 2D [14–17]. However, the role of EGF-mediated anterograde lysosome trafficking in 3D invasion and protease secretion was never investigated. To test this, we performed 3D–Matrigel invasion assays in the presence of DQ-collagen IV, a dye-quenched collagen that fluoresces upon proteolytic cleavage [22, 43]. DU145 cells were grown on a matrix of DQ-collagen IV and Matrigel and incubated with the p38 inhibitor SB203580, the NHE inhibitor EIPA, or vehicle control in the presence or absence of EGF. Cells were fixed and stained for actin (red) and imaged using confocal microscopy. Green represents cleaved DQ-collagen IV as a readout for protease activity (Fig. 6b; quantified in Fig. 6c). Cells grown in the absence of EGF form spheroid-like colonies with minimal cleaved DQ-collagen IV fluorescence. Cells treated with EGF exhibit a more invasive phenotype characterized by the loss of spheroid colony morphology and the appearance of cellular protrusions. This invasive morphology was accompanied by increased DQ-collagen IV fluorescence (green) indicating increased protease secretion and activity. EGF-driven invasive morphology and protease activity was reduced in the presence of SB203580 and EIPA. Collectively, these results indicate that anterograde lysosome trafficking occurs in a more physiologically relevant culture model and that lysosome trafficking contributes to the invasive and proteolytic phenotype of EGF-stimulated cells grown in 3D culture.

## Discussion

The present study defines a role for anterograde lysosome trafficking as a necessary event for EGF-mediated protease secretion and tumor cell invasion in DU145 cancer cells. EGF stimulation induced anterograde lysosome trafficking in both 2D and 3D cultures, and EGF-mediated lysosome trafficking is controlled by NHE activity and p38 MAPK signaling. Importantly, inhibition of anterograde lysosome trafficking prevents EGF-mediated invasion through Matrigel in the context of transwell assays and 3D culture, highlighting the importance of lysosome trafficking in cancer invasion.

RTKs, including EGFR and c-Met, share many of the same downstream signaling pathways. Although both EGFR and c-Met activation drive scattering and lysosome trafficking, these two RTKs appear to do so via different intracellular signaling mechanisms. We found that EGF-mediated anterograde lysosome trafficking was regulated in part by the activity of NHEs, similar to the lysosome trafficking events induced by HGF stimulation. However, while HGF required signaling through PI3K and ERK, EGF-induced anterograde lysosome trafficking and protease secretion required signaling through p38 MAPK (Figs. 4 and 6) [17]. It is interesting that c-Met and EGFR require different downstream signaling events for the initiation of a similar lysosome trafficking phenotype as these two RTKs stimulate cell proliferation and invasion, share many of the same downstream signaling pathways, and can even transactivate one another [31–33]. One might predict that both RTKs would use similar downstream signaling events to stimulate organelle movement, protease secretion, and motility. The identification of this new signaling cascade promoting lysosome movement highlights the complex and divergent mechanisms involved in anterograde lysosome trafficking and that different external stimuli induce lysosome trafficking by various internal cellular signaling mechanisms. The development of multiple signaling pathways leading to the same phenotypic outcome may be a survival mechanism allowing tumor cells to overcome anti-cancer treatments, leading to drug resistance. Thus, in spite of recent advances that appreciate the intricacies of cell signaling, much remains to be learned in order to effectively develop targeted therapies. In fact, our lab and many others have previously demonstrated that c-Met/EGFR can compensate for each other when the other is pharmacologically inhibited [44, 45]. Thus, the fact that the induction of lysosome trafficking differs between HGF/c-Met and the EGF/EGFR signaling initiation, suggests that future clinical inhibition of lysosome trafficking may have to inhibit multiple upstream signaling pathways or a common downstream target.

We observed that NHE activity was necessary for EGF-mediated lysosome trafficking and invasion, but not overall cell motility (Fig. 3). Both EGFR and NHEs are overexpressed or hyper-activated in many invasive cancers [4, 46]. While both of these cell surface proteins independently influence tumor growth, their activation state may be coupled. In support of this, Cardone et al. recently found that EGFR forms a complex with NHE1 in pancreatic ductal carcinoma [47]. Indeed, EGF stimulation is known to activate NHE1 through Janus kinase and calmodulin signaling [36, 37] and recently the transcription factor Zeb1 has been reported to control lysosome trafficking resulting in increased cell invasiveness [48]. Also, NHE1 contains a cytoplasmic tail containing an ezrin-rodoxin-moesin (ERM) domain that associates with proteins that regulate actin polymerization [49, 50]. Through interaction with the actin cytoskeleton, NHE activity may facilitate EGFR-mediated motility and invadopodia formation. As lysosomes traffic along microtubules and actin filaments, it stands to reason that NHEs regulate lysosome positioning through control of cytoskeletal components. In support of this, previous studies identified RhoA, a major regulator of actin dynamics, as a mediator of acidic extracellular pH and HGF-driven anterograde lysosome trafficking [16, 17, 51, 52]. Additionally, NHE-mediated proton extrusion functions to acidify the nearby extracellular environment. Many proteases, including lysosomal cathepsins and MMPs, function optimally at acidic pH and their extracellular activity may be enhanced by NHE activity [53, 54]. Thus, regulation of lysosome trafficking, extracellular protease activity, and cytoskeletal rearrangements suggest that NHEs promote tumor growth through multiple mechanisms. Lastly, the trafficking of lysosomal membrane proteins to the plasma membrane has been reported to protect tumor cells from microenvironmental acidosis-induced cell death [55]. Whether this increase in lysosomal membrane proteins within the cell membrane is due to lysosome anterograde trafficking and subsequent fusion remains to be determined.

EGFR activation leads to a myriad of downstream signaling events, many of which promote cellular survival, proliferation, and motility. The p38 MAPK is activated in response to EGFR signaling and we found that pharmacological inhibition of p38 α/β prevented EGF-mediated anterograde lysosome trafficking, protease secretion, cell scattering, and invasion (Figs. 1, 4 and 6). p38 MAPK is phosphorylated by the upstream MKK3/6 [56, 57] and regulates cell motility through a variety of mechanisms including down regulation of E-cadherin [58] and activation of Rho family proteins [59–61]; these may be the mechanisms by which p38 regulates EGF-mediated cell motility and lysosome trafficking. Contrary to our findings, p38 MAPK is reported to directly phosphorylate kinesin-1, resulting in the inhibition of kinesin-1-mediated transport [62]. Kinesin-1 is a reported major driver of anterograde lysosome distribution [13, 63]. While our model of EGF stimulated lysosome trafficking does not fit with this previously defined p38-mediated regulation of kinesin-based transport, there are many other possible levels of control of anterograde organelle movement. For example, ADP-ribosylation factor-like 8b (Arl8b), is a GTPase that recruits kinesin1 to lysosomes, thereby controlling lysosome positioning [63, 64] has been recently reported to control lysosome trafficking downstream of c-Met and EGFR [65]. It is not known whether p38 regulates the activity of Arl8b or the currently unidentified guanine nucleotide exchange factors (GEFs) and GTPase activating proteins (GAPs), which dictate Arl8b function or if a different kinesin protein complex is involved. Future studies should aim to identify the precise mechanisms by which p38 MAPK regulates EGF-mediated anterograde lysosome trafficking.

We found that lysosomes traffic to actin rich cellular protrusions of invasive EGF treated cells grown in 3D culture. These cellular protrusions may be invadopodia, or actin rich invasive “feet” found in cells invading through the ECM. This hypothesis is supported by previous findings that cathepsin B rich LAMP-1 positive vesicles traffic to invadopodia and facilitate in ECM breakdown [18]. Unexpectedly, we also observed lysosomes close to the cell periphery in 3D culture in the absence of EGF stimulation (Fig. 6a). However, there were no invadopodia-like structures observed under control treatment conditions. This suggests that cell invasion and invadopodia formation is regulated by mechanisms more complex than simply lysosome proximity to the plasma membrane. This does provoke the question of whether lysosomes are recruited to the plasma membrane before or after the initiation of invadopodia. Invadopodia are considered mature when they acquire proteoloytic activity [66], suggesting that invadopodia are formed first followed by the recruitment of lysosomes. Additionally, Sung et al. found that cortactin co-localizes with LAMP-1 positive Rab7 positive vesicles and that anterograde trafficking is necessary for the formation of lamellipodia in migrating cells [67]. Cortactin is a critical component of the invadopodia and some evidence supports the recruitment of LAMP-1 positive vesicles as a regulator of leading edge actin dynamics and possibly invadopodia formation. However, the data presented herein suggests that lysosome proximity to the plasma membrane is necessary, but not sufficient to drive invasive behavior in cells grown in a 3D matrix. There may be a rearrangement or thinning of the cortical actin network at sites of invadopodia formation that allows lysosomes to fuse with the plasma membrane in invasive cells. Similar dynamics are observed at the immunological synapse in the process of degranulation of cytotoxic T lymphocytes [68]. It is likely that additional signaling events are required for lysosome fusion with the plasma membrane and subsequent tumor invasion. Although this paper establishes a role of p38 in EGF-induced lysosome trafficking and invasion in two different cell lines, many outstanding questions remain to be answered regarding the mechanism and role of lysosome trafficking in cell invasion.

Clinically, EGFR kinase inhibitors and blocking antibodies are available; however, resistance to these anti-tumor therapies is common, leading to highly invasive and metastatic tumor outgrowth [69]. Thus, the identification of the molecular mechanisms that govern EGF-mediated invasion is of critical importance in order to identify novel anti-cancer targets, and this study suggests that preventing anterograde lysosome trafficking is a potentially viable and potent therapeutic target of EGF-driven tumors.

## Conclusions

This study demonstrates for the first time that EGF can stimulate lysosome trafficking to the cell periphery and that EGF-induced lysosome trafficking is necessary for protease secretion and tumor cell invasion medicated in part through p38 MAPK activation.

## Additional files

Additional file 1: Figure S1. Quantification of western blot data from Fig. 2c and d. ImageJ software was used to perform densitometry analysis on the western blot data. Relative intensity ratios of the protein detected to tubulin was used to determine quantified levels of each protein. Each bar corresponds to a protein band and lane on the western blot in Fig. 2c and d. *indicates significant inhibition (*p* < 0.05) of phosphorylation compared to their respective growth factor stimulated condition (EGF or HGF). **indicates that EGF does not significantly activate pMet (*p* < 0.05; each EGF treatment versus HGF) at any time period. (TIFF 617 kb)

Additional file 2: Figure S2. Early Endosomes, mitochondria, and Golgi body do not display altered positioning upon treatment with EGF. DU145 cells were stimulated with 100 ng/mL EGF for 16 h then fixed and stained for EEA1 (Early Endosomes) and GM130 (Cis Golgi). Mitotracker (mitochondria) was loaded into live cells 30 min prior to fixation. Scale bar represents 30 μm, *N* = 3. (TIFF 165 kb)

Additional file 3: Figure S3. NHE and p38 inhibition block EGF-mediated anterograde lysosome trafficking in HeLa cells. HeLa cells were pre-treated for 2 h with DMSO, 25 μM EIPA, or 10 μM SB203580 then stimulated with 100 ng/mL EGF for 16 h. Cells were fixed and stained for LAMP-1 (red), actin (green), and DAPI (blue). White arrows indicate LAMP-1 positive vesicles in actin rich protrusions. Scale bar represents 30 μm, *N* = 3. (TIFF 229 kb)

Additional file 4: Figure S4. Quantification of western blot data from Fig. 4a. ImageJ software was used to perform densitometry analysis on the western blot data. Relative intensity ratios of the protein detected to tubulin was used to determine quantified levels of each protein. Each bar corresponds to a protein band and lane on the western blot in Fig. 4a. (TIFF 448 kb)

Additional file 5: Figure S5. p38 signaling and not JAK, JNK, or NFkB signaling is necessary for EGF-mediated lysosome trafficking. (A) Cells were treated with 10 μM of the indicated p38 inhibitors or inactive analog (SB202474) for 2 h followed by stimulation with 100 ng/mL EGF for 16 h. Cells were then fixed and stained for LAMP-1 (red), phalloidin (green), and DAPI (blue). Arrows indicate lysosomes at cell periphery. Scale bar represents 30 μm, *N* = 3. (B) Cells were treated with 10 μM of the indicated inhibitors for 30 min prior to stimulation with 100 ng/mL EGF for 10 min. Whole cell lysates were collected and probed for the indicated proteins by western blot (left). Densitometry analysis was performed on the western blot using ImageJ software. *indicates statistically significant phosphorylation of p38 by EGF (p < 0.05). (C) Cells were treated with 10 μM of inhibitors JAK (AG490), JNK (SB600125), and NFkB (Bay11) for two hours followed by stimulation with 100 ng/mL EGF for 16 h. Cells were then fixed and stained for LAMP-1 (red), phalloidin (green), and DAPI (blue), *N* = 3. Scale bar represents 30 μm. (TIFF 1056 kb)

Additional file 6: Figure S6. Quantification of western blot data from Fig. 5A. ImageJ software was used to perform densitometry analysis on the western blot data. Relative intensity ratios of the protein detected to tubulin was used to determine quantified levels of each protein. Each bar corresponds to a protein band and lane on the western blot in Fig. 5a. (TIFF 406 kb)

## Abbreviations

Arl8b: ADP ribosylation factor like 8b
ECM: Extracellular matrix
EGF: Epidermal growth factor
EGFR: Epidermal growth factor receptor
EIPA: 5-(N-ethyl-N-isopropyl-Amiloride
EMT: Epithelial to mesenchymal transition
ERK: Extracellular signal-regulated kinase
HGF: Hepatocyte growth factor
LAMP-1: Lysosome associated membrane protein-1 cancer
MAPK: Mitogen activated protein kinase
MTOC: Microtubule organizing center
NHE: Sodium hydrogen exchanger
RTK: Receptor tyrosine kinase
Tro: Troglitazone
