# Supplementary figures and images for: Lysosome trafficking is necessary for EGF-driven invasion and is regulated by p38 MAPK and Na+/H+ exchangers
Source: BMC Cancer. 2017 Oct 4;17:672. doi: 10.1186/s12885-017-3660-3 (PMC5628462; doi:10.1186/s12885-017-3660-3)

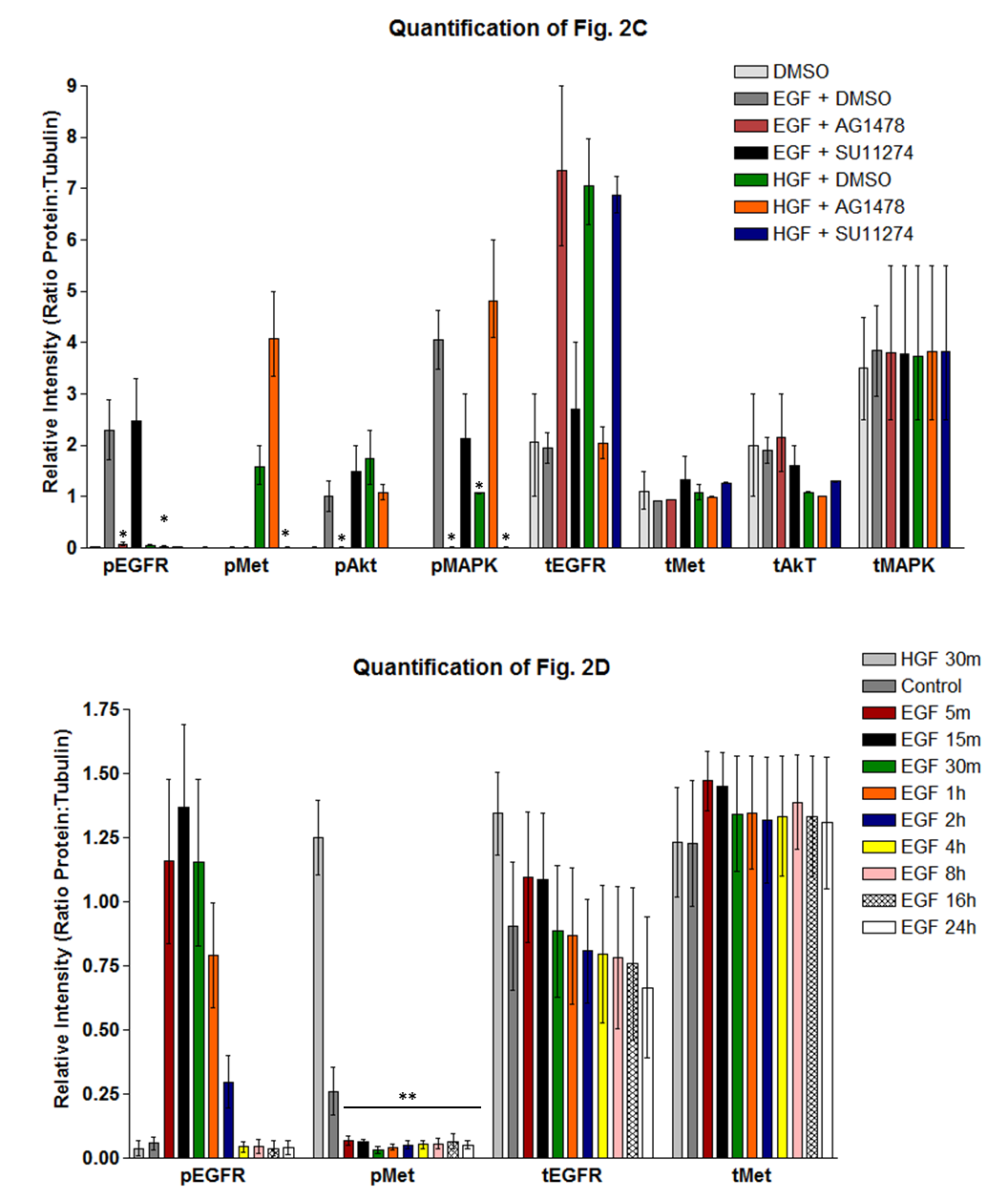

Supplement: Supplementary file 1 — Quantification of western blot data from Fig. 2c and d. ImageJ software was used to perform densitometry analysis on the western blot data. Relative intensity ratios of the protein detected to tubulin was used to determine quantified levels of each protein. Each bar corresponds to a protein band and lane on the western blot in Fig. 2c and d. *indicates significant inhibition (p < 0.05) of phosphorylation compared to their respective growth factor stimulated condition (EGF or HGF). **indicates that EGF does not significantly activate pMet (p < 0.05; each EGF treatment versus HGF) at any time period. (TIFF 617 kb) [file 12885_2017_3660_MOESM1_ESM.tif]

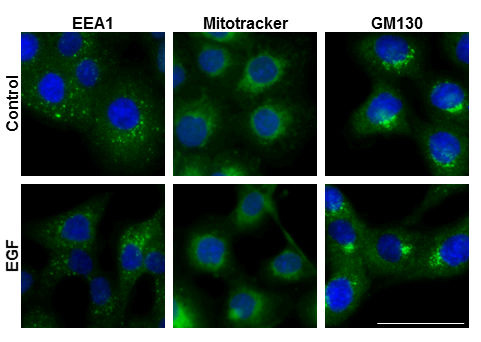

Supplement: Supplementary file 2 — Early Endosomes, mitochondria, and Golgi body do not display altered positioning upon treatment with EGF. DU145 cells were stimulated with 100 ng/mL EGF for 16 h then fixed and stained for EEA1 (Early Endosomes) and GM130 (Cis Golgi). Mitotracker (mitochondria) was loaded into live cells 30 min prior to fixation. Scale bar represents 30 μm, N = 3. (TIFF 165 kb) [file 12885_2017_3660_MOESM2_ESM.tif]

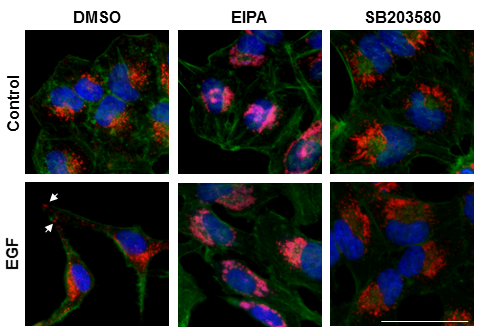

Supplement: Supplementary file 3 — NHE and p38 inhibition block EGF-mediated anterograde lysosome trafficking in HeLa cells. HeLa cells were pre-treated for 2 h with DMSO, 25 μM EIPA, or 10 μM SB203580 then stimulated with 100 ng/mL EGF for 16 h. Cells were fixed and stained for LAMP-1 (red), actin (green), and DAPI (blue). White arrows indicate LAMP-1 positive vesicles in actin rich protrusions. Scale bar represents 30 μm, N = 3. (TIFF 229 kb) [file 12885_2017_3660_MOESM3_ESM.tif]

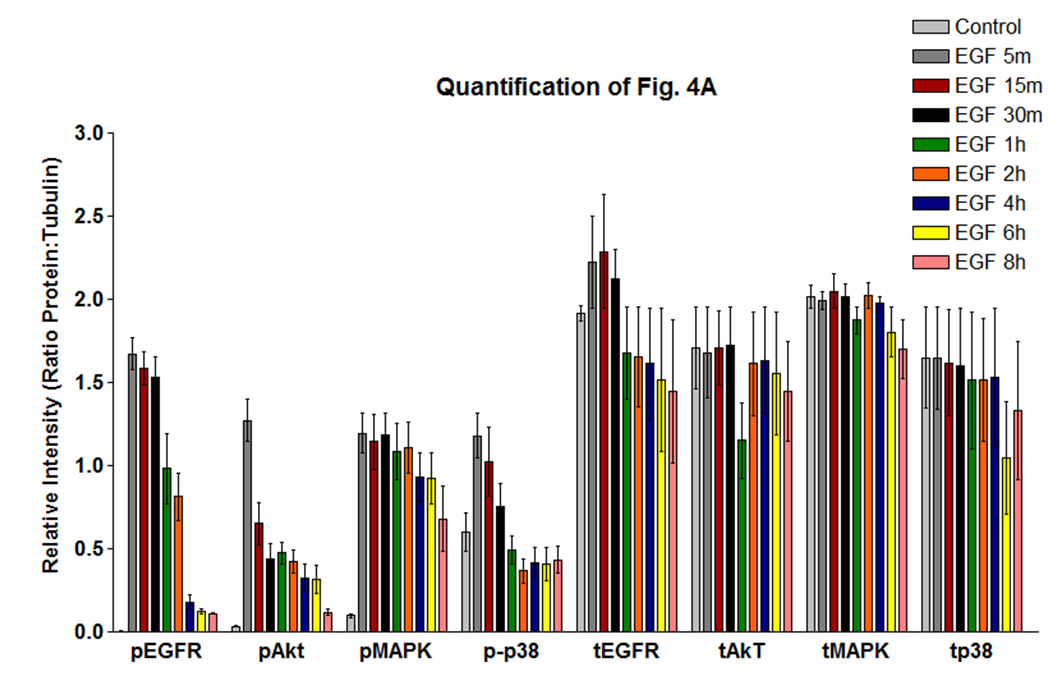

Supplement: Supplementary file 4 — Quantification of western blot data from Fig. 4a. ImageJ software was used to perform densitometry analysis on the western blot data. Relative intensity ratios of the protein detected to tubulin was used to determine quantified levels of each protein. Each bar corresponds to a protein band and lane on the western blot in Fig. 4a. (TIFF 448 kb) [file 12885_2017_3660_MOESM4_ESM.tif]

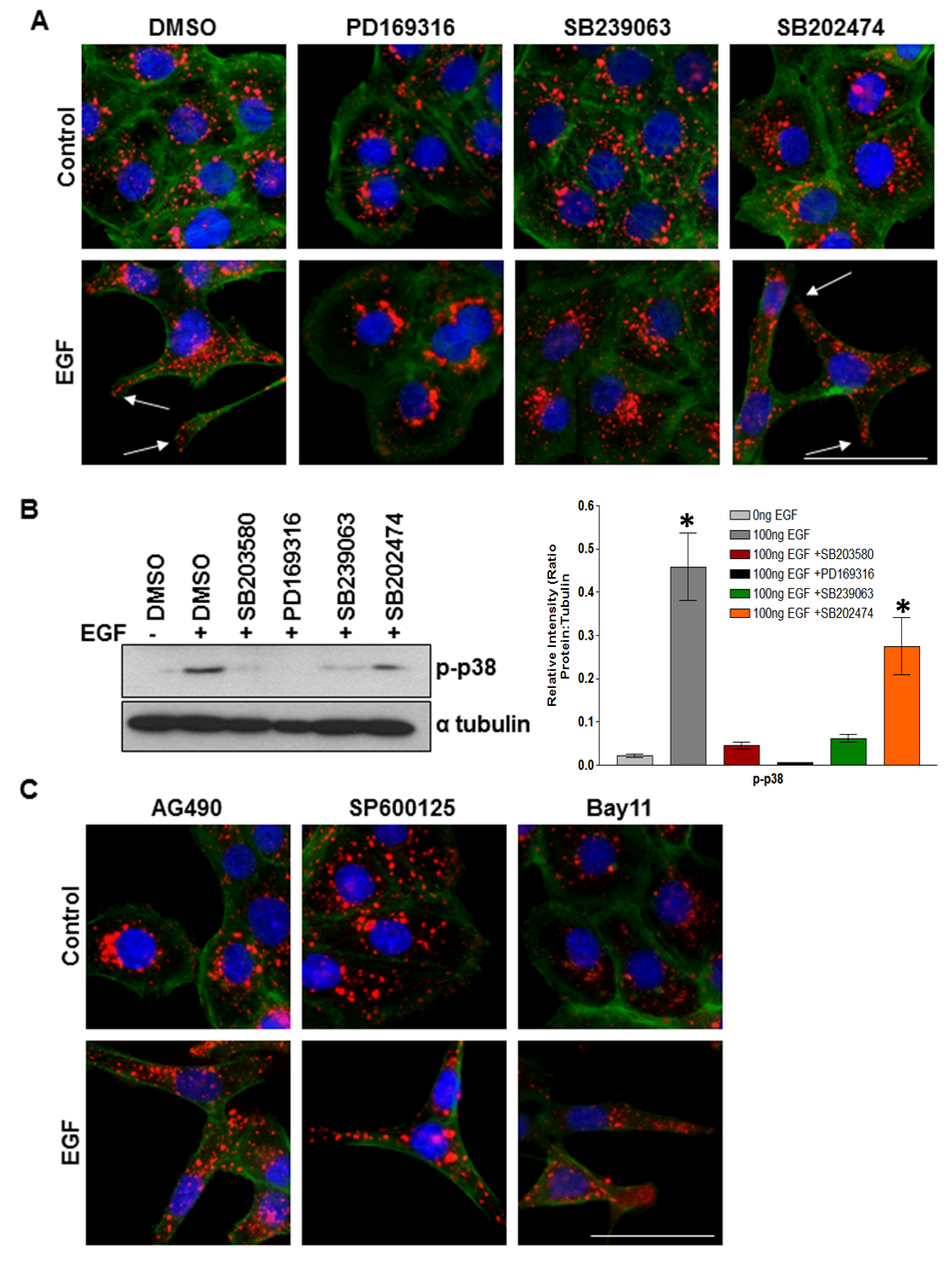

Supplement: Supplementary file 5 — p38 signaling and not JAK, JNK, or NFkB signaling is necessary for EGF-mediated lysosome trafficking. (A) Cells were treated with 10 μM of the indicated p38 inhibitors or inactive analog (SB202474) for 2 h followed by stimulation with 100 ng/mL EGF for 16 h. Cells were then fixed and stained for LAMP-1 (red), phalloidin (green), and DAPI (blue). Arrows indicate lysosomes at cell periphery. Scale bar represents 30 μm, N = 3. (B) Cells were treated with 10 μM of the indicated inhibitors for 30 min prior to stimulation with 100 ng/mL EGF for 10 min. Whole cell lysates were collected and probed for the indicated proteins by western blot (left). Densitometry analysis was performed on the western blot using ImageJ software. *indicates statistically significant phosphorylation of p38 by EGF (p < 0.05). (C) Cells were treated with 10 μM of inhibitors JAK (AG490), JNK (SB600125), and NFkB (Bay11) for two hours followed by stimulation with 100 ng/mL EGF for 16 h. Cells were then fixed and stained for LAMP-1 (red), phalloidin (green), and DAPI (blue), N = 3. Scale bar represents 30 μm. (TIFF 1056 kb) [file 12885_2017_3660_MOESM5_ESM.tif]

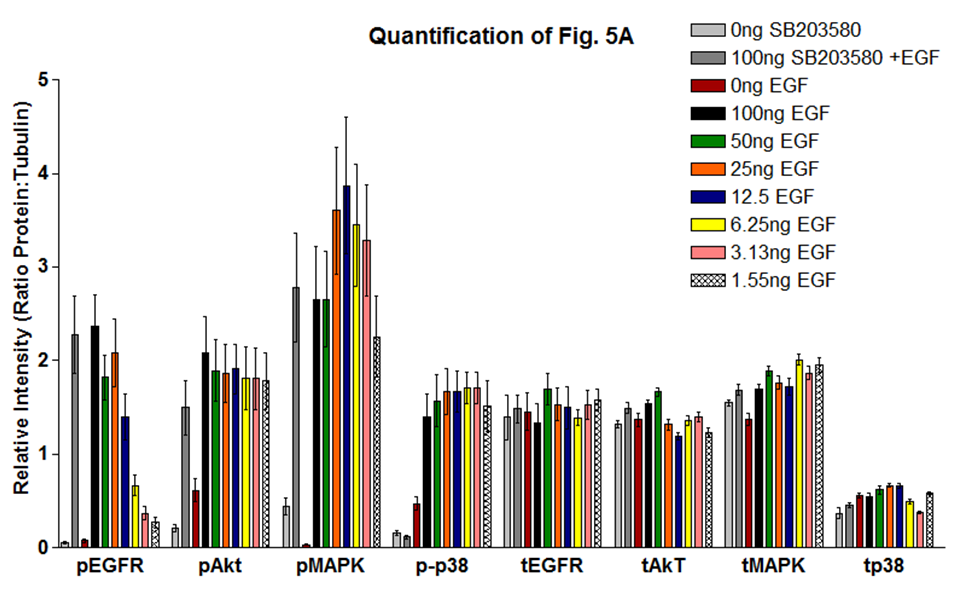

Supplement: Supplementary file 6 — Quantification of western blot data from Fig. 5A. ImageJ software was used to perform densitometry analysis on the western blot data. Relative intensity ratios of the protein detected to tubulin was used to determine quantified levels of each protein. Each bar corresponds to a protein band and lane on the western blot in Fig. 5a. (TIFF 406 kb) [file 12885_2017_3660_MOESM6_ESM.tif]
